# Effects of very low volume high intensity versus moderate intensity interval training in obese metabolic syndrome patients: a randomized controlled study

**DOI:** 10.1038/s41598-021-82372-4

**Published:** 2021-02-02

**Authors:** Dejan Reljic, Fabienne Frenk, Hans J. Herrmann, Markus F. Neurath, Yurdagül Zopf

**Affiliations:** 1 Hector-Center for Nutrition, Exercise and Sports, Department of Medicine 1, University Hospital Erlangen, Friedrich-Alexander University Erlangen-Nürnberg, Ulmenweg 18, 91054 Erlangen, Germany; 2 Department of Medicine 1, University Hospital Erlangen, Friedrich-Alexander University Erlangen-Nürnberg, Erlangen, Germany

**Keywords:** Diseases, Risk factors

## Abstract

Physical activity is a cornerstone in the treatment of obesity and metabolic syndrome (MetS). Given the leading physical activity barrier of time commitment and safety concerns about vigorous exercise in high-risk groups, this study aimed to investigate the effects of two extremely time-efficient training protocols (< 30 min time effort per week), either performed as high- (HIIT) or moderate-intensity interval training (MIIT) over 12 weeks, in obese MetS patients. In total, 117 patients (49.8 ± 13.6 years, BMI: 38.2 ± 6.2 kg/m^2^) were randomized to HIIT (n = 40), MIIT (n = 37) or an inactive control group (n = 40). All groups received nutritional counseling to support weight loss. Maximal oxygen uptake (VO_2max_), MetS severity (MetS z-score), body composition and quality of life (QoL) were assessed pre-and post-intervention. All groups significantly reduced body weight (~ 3%) but only the exercise groups improved VO_2max_, MetS z-score and QoL. VO_2max_ (HIIT: + 3.1 mL/kg/min, *p* < 0.001; MIIT: + 1.2 mL/kg/min, *p* < 0.05) and MetS z-score (HIIT: − 1.8 units, *p* < 0.001; MIIT: − 1.2 units, *p* < 0.01) improved in an exercise intensity-dependent manner. In conclusion, extremely low-volume interval training, even when done at moderate intensity, is sufficiently effective to improve cardiometabolic health in obese MetS patients. These findings underpin the crucial role of exercise in the treatment of obesity and MetS.

## Introduction

The worldwide prevalence rates of obesity have continued to rise over the last decades^1^. Obesity is defined as excess body weight with an abnormally high accumulation of body fat that is associated with an increased risk of several chronic diseases^2^. The clustering of additional cardiometabolic risk factors along with obesity, such as excess abdominal fat, hypertension, dyslipidemia or hyperglycemia (also referred to as the metabolic syndrome, MetS), further increases the risk of serious health conditions, including cardiovascular disease (CVD), various types of cancer and premature mortality^3,4^. Moreover, recent research suggests that obesity and existing cardiometabolic disorders may substantially increase the likelihood of a severe clinical course of acute viral infections, as currently observed among patients with the novel coronavirus disease 2019 (COVID-19)^5,6^. Consequently, the development and evaluation of effective and feasible treatment strategies for obesity and related disorders is probably more topical than ever.

Adequate dietary changes and physical exercise are crucial components in the treatment of obesity and associated comorbidities. It has been demonstrated, for example, that men and women who are overweight/obese but physically active display a lower risk of morbidity and mortality than individuals who are inactive^7^. More specifically, the degree of cardiorespiratory fitness (CRF, typically expressed as maximal oxygen uptake, VO_2max_) has been shown to be an independent predictor of CVD and mortality—stronger than other established risk factors, such as smoking, hypertension or increased body mass index (BMI)^8^. Nevertheless, despite vast evidence of the health benefits of regular physical activity (PA) and exercise and the negative consequences of a sedentary lifestyle, most obese individuals are not meeting the recommended minimum levels of PA (i.e. 150 min of moderate or 75 min of vigorous PA throughout the week)^9^. The underlying reasons why obese individuals do not participate in regular PA are manifold and complex. However, as in the general population, the most commonly cited barrier to adopting and maintaining a more physically active lifestyle is “a perceived lack of time”^10^. Moreover, it has been suggested that the widespread approach of presenting 150 min/week of PA as a necessary minimum threshold for achieving health benefits may have negative impacts on motivation because it is not a realistic target for most adults^11^.

In this context, high‐intensity interval training (HIIT) has emerged as an attractive, more time-efficient exercise option to higher-volume moderate-intensity continuous training (MICT), which has been the most commonly prescribed exercise modality in weight loss programs for the past decades. HIIT is a type of cardiovascular training that typically involves brief intense bouts of exercise at intensities of ≥ 80% of maximal heart rate (HR_max_) separated by recovery periods of low-intensity activity or rest^12^. A number of meta-analyses have shown that HIIT can improve CRF^13–16^, body composition^15,17–19^ and various cardiometabolic risk markers^13,14,16^ in overweight/obese individuals and patients with cardiometabolic diseases effectively within only a few weeks. Recently, it has also been demonstrated that the uptake of HIIT after bariatric surgery may contribute to prevent weight regain and stabilize cardiometabolic risk profile in obese patients^20^.

“Low-volume” HIIT and sprint interval training (SIT) are particularly time-efficient subtypes of interval training, which have gained increasing attention in recent years^21,22^. As per previous definition, these brief training protocols typically involve ≤ 10 min of intense exercise within a session lasting ≤ 30 min including warm-up, recovery phases between intervals and cool-down^21^. There is accumulating evidence that these low-volume exercise approaches can induce similar or even superior improvements in CRF than MICT, despite substantially lower training volume and time commitment^23–25^. However, the majority of this evidence is based on studies involving trained, sedentary or overweight but otherwise healthy participants. To date, far less is known about the efficacy of low-volume HIIT to improve CRF and cardiometabolic risk markers in clinical populations, such as obese MetS patients, who represent a particular high-risk group for cardiac adverse events^26^. Ramos et al.^27^ have demonstrated in a pioneering study that low-volume HIIT (one vigorous 4-min bout at 85%–95% HR_peak_ within an exercise session of 17 min total duration, including warm-up and cool-down), performed three times weekly (51 min total time effort per week) over a period of 16 weeks, induced significant improvements in CRF and cardiometabolic health in obese MetS patients. Importantly, it was reported that there were no adverse events that were related to the exercise intervention. Moreover, some studies have demonstrated that low-volume HIIT protocols with weekly exercise volumes in the range of 57 to 113 min and exercise intensities ranging from 90 to 100% HR_max_, respectively, improved glucose control in obese type 2 diabetes patients^28–31^.

However, given the leading exercise barrier of time commitment, there is need to assess the efficacy of even more time-efficient low-volume HIIT protocols on health outcomes in obese individuals at increased cardiometabolic risk. In addition, given remaining adherence^32^ and health^26,33^ concerns about vigorous exercise in previously sedentary individuals, it should be elucidated whether a simultaneous reduction in the exercise volume and intensity (i.e. low-volume moderate-intensity interval training) would still be effective at inducing physiological adaptations that are linked to improved health outcomes.

The aim of the present study was, therefore, to investigate the effects of two extremely time-efficient interval training protocols (< 30 min time effort per week), either performed as high-intensity (≥ 80% HR_max_, HIIT), which was previously shown to induce similar improvements in CRF compared to higher-volume MICT in sedentary, normal-weight individuals^34^, or moderate-intensity interval training (≤ 80% HR_max_, MIIT) versus an inactive control group (CON) on VO_2max_ and cardiometabolic risk profile (quantified by the metabolic syndrome z-score, MetS z-score) in obese MetS patients. We hypothesized that both protocols would improve VO_2max_ and MetS z-score in this particular risk group when compared to CON, but that health benefits induced by HIIT would be superior to MIIT.

## Methods

### Study design

This study was a 12-week randomized-controlled trial (ClinicalTrials.gov Id: NCT03306069. Registered 10 October 2017, https://clinicaltrials.gov/ct2/show/NCT03306069). Participants were randomly assigned to the inactive CON group, only receiving nutritional counseling, or an exercise group (either performing HIIT or MIIT) plus nutritional counseling. Randomization was performed employing a computerized random number generator (MinimPy, GNU GPL v3), independently of the researchers who were involved in data collection. Primary outcome was VO_2max_, secondary outcomes were MetS z-score, body composition and self-reported quality of life (QoL). Participants were fully informed about the aims and procedures of the study, which conformed to the Helsinki Declaration. All participants provided written consent before the onset of study procedures. The study protocol was approved by the Medical Ethical Committee of the Friedrich-Alexander University Erlangen-Nürnberg (approval number: 210_17B).

### Participants

Participants were recruited through local newspaper advertisements. Inclusion criteria were: age ≥ 18 years, obesity (BMI ≥ 30 kg/m^2^) and increased waist circumference (> 88 cm for females, > 102 cm for males) plus at least two additional cardiometabolic abnormalities, including hypertension (≥ 130 mmHg systolic and/or ≥ 85 mmHg diastolic blood pressure), dyslipidemia (triglycerides: ≥ 150 mg/dL; high-density lipoprotein cholesterol [HDL)]: < 50 mg/dL for females, < 40 mg/dL for males) and hyperglycemia (≥ 100 mg/dL)^35^, and a self-reported sedentary lifestyle^36^. Exclusion criteria were: clinical diagnosis of heart disease, cancer, severe orthopaedic conditions or other major health problems that might preclude safe participation in exercise and pregnancy. Participants agreed not to change their medications or dosages without medical consultation and informing the principal investigator and to maintain their usual lifestyle patterns throughout the study to minimize potential confounding effects. In addition, participants who did not attend at least 75% of exercise sessions were excluded from the study.

Based on previously published data^34^, suggesting a large effect (*d* = 0.97) on the primary outcome (relative VO_2max_), a priori sample size calculation indicated that n = 16 per group would be required to yield a power of 0.95 in a 2-sided ANOVA with a 5% level of significance (G*Power, version 3.1.9.2). However, given that the literature reports attrition rates in obesity interventions of up to 80%^37^, we aimed to recruit a minimum of n = 25 per group to sufficiently account for dropouts.

### Health examination

All procedures were carried out under laboratory conditions in a stable ambient environment at our Research Center as previously described elsewhere^38^. Baseline examinations were carried out 1 week pre-intervention. Follow-up examinations were conducted within the first week post-intervention, at least 3 days apart from the last training session and at a similar time of day to allow sufficient recovery and to avoid possible circadian effects. Participants were advised to arrive overnight-fasted and to refrain from alcohol and vigorous PA for at least 24 h prior to examinations. The assessments were carried out in a single-blinded fashion, meaning that the investigators who collected the data were not aware of the participants’ group assignment.

### Blood pressure measurements

Participants were asked to empty their bladder before the measurements were conducted. Subsequently, participants rested in a seated position for 5 min before blood pressure values were measured using an automatic upper-arm blood pressure monitor (M5 professional, Omron, Mannheim, Germany), which has been validated for accuracy^39^. Two consecutive measurements on both arms were obtained at 60 s intervals and the averaged values of the arm with the higher pressure were used in the analysis as previously recommended^40^.

### Blood sampling

Blood samples were drawn via antecubital venipuncture into different collection tubes using a disposable cannula (S-Monovette, Sarstedt, Nürmbrecht, Germany) in a standardized manner^38^. The collected blood samples were analyzed in the laboratories of the University Hospital Erlangen. Serum concentrations of glucose, total-cholesterol, low-density lipoprotein cholesterol (LDL), high-density lipoprotein cholesterol (HDL) and triglycerides were determined by a photometric method (Clinical Chemistry Analyzer AU700/AU5800, Beckman Coulter, Brea, CA, USA), with average coefficients of variation (CV) ranging from 1.1 to 1.4%. Serum glycosylated hemoglobin A_1c_ (HbA_1c_) was measured by means of a turbidimetric immuneassay (COBAS Integra 400, Roche Diagnostics, Mannheim, Germany, CV: 2.7%).

### Anthropometric and body composition measurements

Waist circumference was measured with a measuring tape while participants were in a standing position. Body composition measurements were conducted using a segmental multi-frequency bioelectrical impedance analysis device (seca mBCA 515, Seca, Hamburg, Germany), which has been shown to provide accurate determination of body composition in obese individuals when compared to the 4-compartement reference method^41^. All measurements were performed according to the manufacturer’s instructions.

### Determination of MetS z-score

The MetS z-score is a continuous risk score assessment that was specifically designed to quantify MetS severity. According to the literature, MetS z-score is more sensitive to identify the patient’s overall cardiometabolic risk status compared to single categorical risk criteria, which may miss to detect clinically meaningful changes if certain cut-off values to move out of a distinctive (“pathologic”) category are not achieved^42^. MetS z-score was calculated according to sex-specific equations based on waist circumference (WC), mean arterial blood pressure (MAB), serum concentrations of fasting serum glucose (GLU), triglycerides (TG), and HDL, as follows^43^:

- Men:
  $$ [({4}0{-}{\text{HDL}})/{9}.0\left] + \right[({\text{TG}}{-}{15}0)/{81}.0\left] + \right[({\text{GLU}}{-}{1}00)/{11}.{3}\left] + \right[({\text{WC}}{-}{1}0{2})/{7}.{7}\left] + \right[({\text{MAB}}{-}{1}00)/{9}.{1}]. $$
- Women:
  $$ \left[ {\left( {{5}0{-}{\text{HDL}}} \right)/{14}.{1}} \right] + \left[ {\left( {{\text{TG}}{-}{15}0} \right)/{81}.0} \right] + \left[ {\left( {{\text{GLU}}{-}{1}00} \right)/{11}.{3}} \right] + \left[ {\left( {{\text{WC}}{-}{88}} \right)/{9}.0} \right] + [\left( {{\text{MAB}}{-}{1}00} \right)/{9}.{1}]. $$

### Cycle ergometer test

Participants performed a standardized ramp exercise test on an electronically braked cycle ergometer (Corival cpet, Lode, Groningen, Netherlands) to determine VO_2max_, maximal power output (W_max_) and HR_max_. Following a 1 min familiarization period, the initial load was set at 50 W and then gradually increased by 1 W every 5 s (i.e. 25 W within 2 min) in female participants and by 1 W every 4 s (i.e. 30 W within 2 min) in male participants, respectively, until volitional exhaustion. Criteria to assume that maximal effort was reached were at least two of the following: a leveling-off of oxygen uptake, maximal respiratory exchange ratio ≥ 1.10, age predicted HR_max_ ≥ 90% (using the equation: 220–age) and maximal rate of perceived exertion ≥ 19^44^. HR was recorded continuously using a 12-lead ECG system (custo cardio 110, custo med, Ottobrunn, Germany). Oxygen uptake was measured with an open-circuit breath-by-breath spiroergometric system (Metalyzer 3B-R3, Cortex Biophysik, Leipzig, Germany), which has been shown to be a reliable instrument for cardiopulmonary exercise testing^45^. The system measures ventilation continuously and simultaneously determines oxygen uptake (electrochemical cell) and carbon dioxide output (infrared analyzer). All measurements were averaged over every 10 s. Additionally, ventilatory threshold (VT) was determined according to the V-slope method by plotting carbon dioxide output (VCO_2_) against oxygen uptake (VO_2_) in order to assess submaximal exercise capacity^46^.

### Assessment of self-reported outcomes

The EQ-5D-5L questionnaire was used to assess health-related QoL. The questionnaire consists of a visual analogue scale (EQ-VAS, 0–100 points, higher values indicate higher QoL) and a descriptive system of 5 health-related QoL-dimensions (mobility, self-care, usual activities, pain/discomfort, anxiety/depression) with 5 severity levels each, which are converted to a single index value (EQ-5D-5L). An index value of 1.0 represents the best possible state of perceived health, while an index value of 0 represents the worst possible health status. The questionnaire was previously validated in the German language^47^. Additionally, participants provided a personal evaluation sheet at the end of the study, including their enjoyment of the intervention on a 7-point rating scale (1 = not enjoyable at all; 7 = extremely enjoyable).

### Nutritional counseling

Participants received nutritional counseling by a registered dietitian in a face-to-face meeting at study entry on how to modify their daily diet at home. As recommended by international guidelines for the prevention and treatment of obesity^48^, participants were advised to achieve a daily energy deficit of 500 kcal to promote weight loss. In addition, handouts for meal planning were provided to participants to support the implementation of dietary recommendations for reduced calorie intake. Nutritional intake was monitored by 24 h-dietary records (Freiburger Ernährungsprotokoll; Nutri-Science, Freiburg, Germany) assessed on 3 consecutive days at study entry and within the last week of the intervention. Computer-based analysis of mean caloric and nutrient intake was done by the software PRODI 6 expert (Nutri-Science, Freiburg, Germany).

### Interval training

Training was performed on electronically braked cycle ergometers (Corival cpet, Lode, Groningen, Netherlands) and supervised by certified physiotherapists/sports therapists. Exercise sessions were conducted twice a week (with at least 1 day rest in between) over a period of 12 weeks. The HIIT protocol was similar to the protocol developed by Reljic et al.^34^. Specifically, the protocol consisted of a 2 min warm-up phase, 5 interval bouts of 1 min at 80–95% HR_max_ interspersed with 1 min of low intensity recovery and a 3 min cool-down phase (total session time: 14 min). The minimum intensity to be achieved was progressively increased every 4 weeks during the intervention (weeks 1–4: 80–85%, weeks 5–8: 85–90%, and weeks 9–12: 90–95% HR_max_, respectively). The MIIT protocol was designed identically (i.e. 2 min warm-up, 5 interval bouts of 1 min interspersed with 1 min of low intensity recovery and 3 min cool-down phase; total session time: 14 min), with the exception that participants were required to achieve an exercise intensity in the range of 65–80% HR_max_ during all sessions. To reach their individual target HR for each interval bout, participants were advised to adjust the pedal cadence and/or increase/decrease load resistance. Participants were provided with a chest strap HR monitor (Polar H7 heart rate sensor, Polar Electro Oy, Kempele, Finland) to continuously track their HR during exercise. Participants’ HR was recorded throughout each exercise session and subsequently, HR responses during each interval were analyzed using a specific HR monitoring system (Polar Team, Polar Electro Oy, Kempele, Finland). Participants were able to schedule their exercise sessions individually during the opening hours of the training center, which allowed a close supervision with an average therapist-participant ratio of 1:2.

### Statistical analysis

All statistical analyses were performed using SPSS version 24.0 (SPSS Inc., Chicago, IL, USA). First, the distribution of data was checked using the Shapiro–Wilk test. A 2 × 2 repeated-measures ANOVA was conducted to test for main effects of group, time and interaction between both factors. Homogeneity of variance was verified with the Levene’s test. When significant main or interaction effects were found, post hoc paired t-tests were performed to determine within-group differences between pre- and post-intervention values and one-way ANOVAs followed by Sidak’s post hoc tests were used to assess between-group differences and to compare pre-post-intervention changes between groups, respectively. In case of non-normally distributed data, log or square root transformation was used and the same analyses were applied to the transformed values. If the transformation did not lead to data normalization (percentage of body fat, QoL-outcomes), the non-parametric Friedman two-way analysis of variance by ranks was conducted, followed by Wilcoxon’s and Mann–Whitney tests for post-hoc comparisons. Effect sizes were calculated using the partial eta-squared (ɳ_p_^2^) for ANOVA and Kendall’s coefficient of concordance (*W*) for the Friedman test. Effect sizes were interpreted as: small ≤ 0.01, medium ≥ 0.06, and large ≥ 0.14 for ɳ_p_^2^; and small ≤ 0,10, medium ≥ 0.30, and large ≥ 0.50 for *W*, respectively^49^. For all analyses, the significance level was set at *p* < 0.05. Data are reported as means ± standard deviation (SD) and pre-/post-intervention changes are presented with 95% confidence intervals (95% CI).

## Results

### Study flow

**Figure 1:**
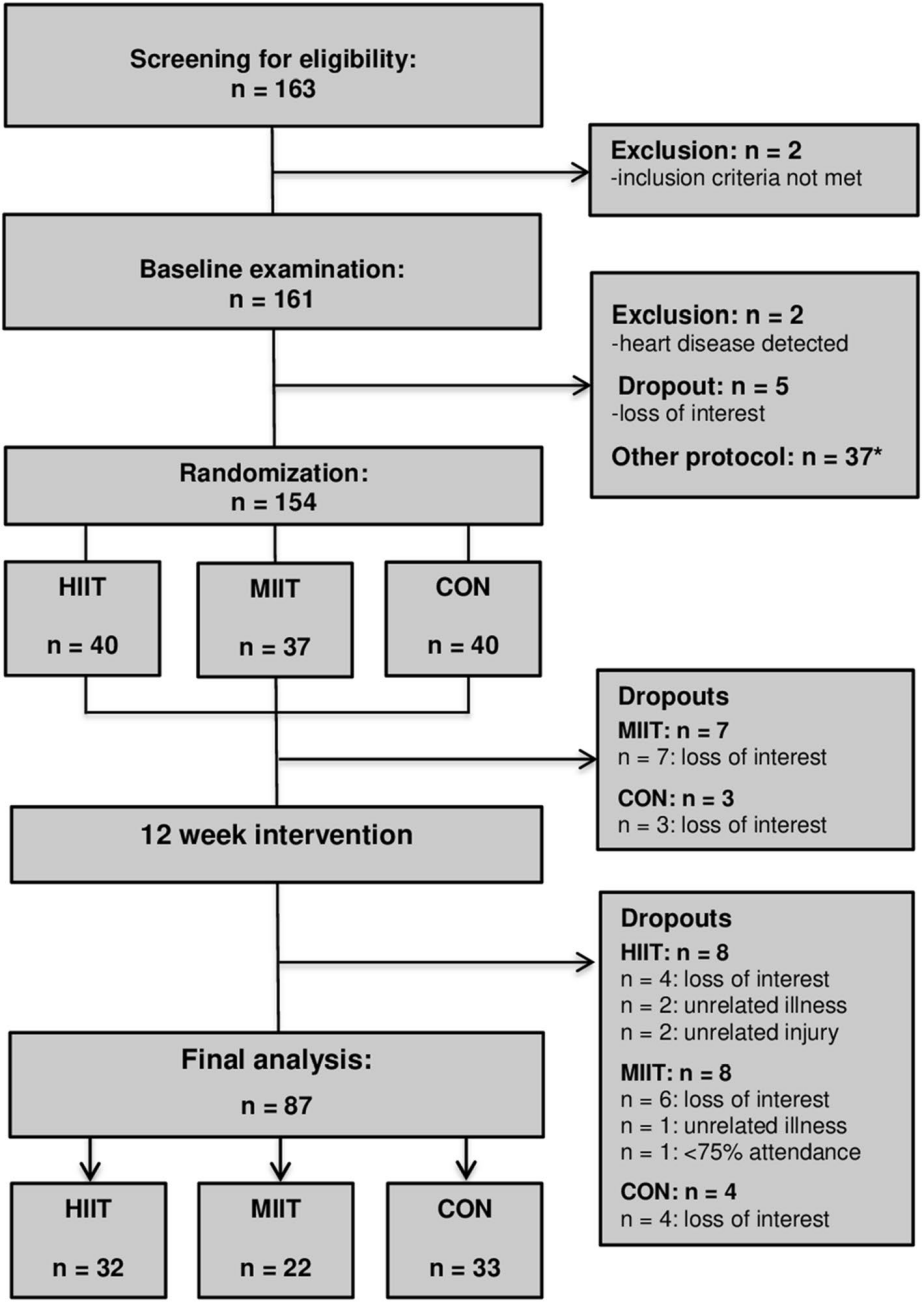
Study flow chart. HIIT, high-intensity interval training group; MIIT, moderate-intensity interval training group; CON, control group. *other exercise group (data not shown because not part of this specific study objective).

A total of 163 individuals were screened for eligibility. Two participants were excluded for not meeting the inclusion criteria, 5 withdrew for personal reasons and 2 were excluded due to medical reasons detected during the health examination. Hence, 154 participants were randomized to either: (i) low-volume high-intensity interval training (HIIT, n = 40), (ii) low-volume moderate-intensity interval training (MIIT, n = 37), (iii) an inactive control group (CON, n = 40), or (iv) another exercise group (n = 37, data not shown because not part of this specific study objective). Table 1 displays the main baseline characteristics of the participants allocated to each group, including their medication. After randomization and before intervention, 10 participants withdrawed from the study because of dissatisfaction with group allocation (MIIT = 7, CON = 3) and during the intervention period, 20 participants dropped out (HIIT = 8, MIIT = 8, CON = 4). The reasons for dropout are displayed in Fig. 1 (Study Flow Chart). A total of 87 participants completed the study and were included in the final analysis (HIIT: n = 32, MIIT: 22, CON: n = 33).

**Table 1:** Baseline characteristics of study participants and medications.

Variable	HIIT (n = 40)	MIIT (n = 37)	CON (n = 40)
Age (years)	49.6 ± 12.3	51.1 ± 15.4	48.8 ± 13.2
Body mass index (kg/m^2^)	38.5 ± 6.4	37.2 ± 6.0	38.7 ± 6.3
VO_2max_ (mL/kg/min)	21.6 ± 5.1	20.2 ± 3.4	20.8 ± 6.9
MetS z-score	3.2 ± 3.7	2.5 ± 3.3	2.0 ± 2.9
**Medications, n (%)**			
Antihypertensives	21 (52.5%)	25 (67.6%)	16 (40%)
Metformin	5 (12.5%)	7 (18.9%)	3 (7.5%)
Exogenous Insulin	1 (2.5%)	1 (2.7%)	1 (2.5%)
Anticoagulants	1 (2.5%)	2 (5.4%)	0
Bronchodilators	1 (2.5%)	1 (2.7%)	2 (5.4%)
Antihistamines	0	0	3 (7.5%)
L-thyroxine	5 (12.5%)	4 (10.8%)	9 (22.5%)
Analgesics	9 (22.5%)	10 (27.0%)	15 (37.5%)
Anti-depressants	5 (12.5%)	8 (21.6%)	5 (12.5%)

*HIIT* high-intensity interval training group, *MIIT* moderate-intensity interval training group, *CON* control group, *VO*_*2max*_ maximal oxygen uptake, *MetS z-score* metabolic syndrome z-score.

### Anthropometric data and body composition

**Figure 2:**
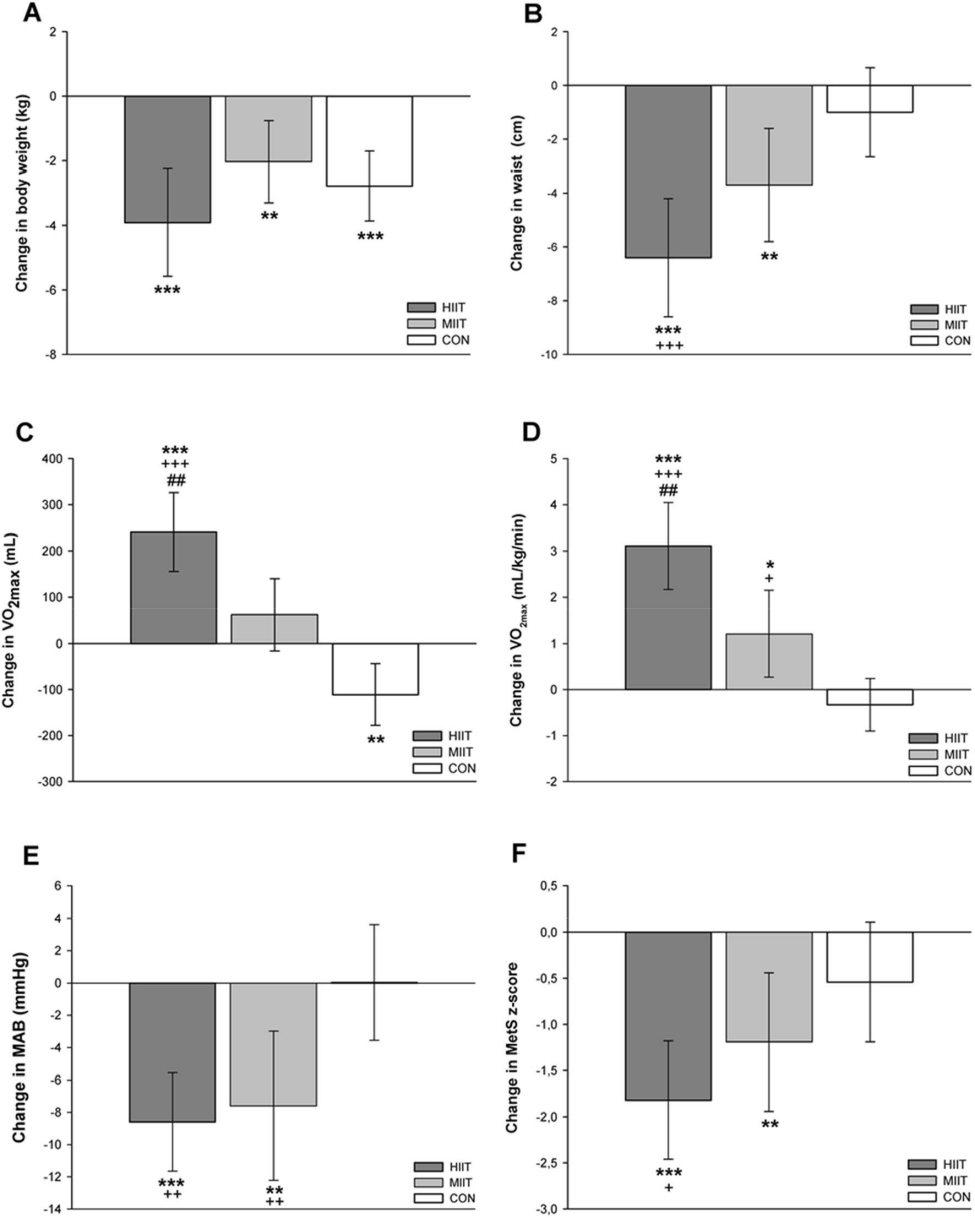
Changes in body weight **(A)**, waist circumference **(B)**, absolute maximal oxygen uptake **(C)**, relative maximal oxygen uptake **(D)**, mean arterial blood pressure **(E)**, and metabolic syndrome z-score **(F)**. HIIT, high-intensity interval training group; MIIT, moderate-intensity interval training group; CON, control group; VO_2max_, maximal oxygen uptake; MAB, mean arterial blood pressure; MetS, metabolic syndrome. *(*p* < 0.05), **(*p* < 0.01), ***(*p* < 0.001), significantly different from pre-intervention. ^+^(*p* < 0.05), ^++^(*p* < 0.01), ^+++^(*p* < 0.001), significant difference vs. CON; ^##^(*p* < 0.01), significant difference vs. MIIT.

Significant main time effects were found for body weight (*p* < 0.001, ή^2^ = 0.40), BMI (*p* < 0.001, ή^2^ = 0.41), fat mass (*p* < 0.001, ή^2^ = 0.30), percentage of body fat (*p* < 0.001, *W* = 0.21), fat free mass (*p* = 0.001, ή^2^ = 0.13), body water (*p* < 0.001, ή^2^ = 0.19) and waist circumference (*p* < 0.001, ή^2^ = 0.34). A significant group-by-time interaction was observed for fat mass (*p* = 0.033, ή^2^ = 0.08) and waist circumference (*p* = 0.001, ή^2^ = 0.15). All three groups achieved a significant reduction of body weight (HIIT: − 3.9 kg, 95% CI: − 5.6 to − 2.2 kg, *p* < 0.001; MIIT: − 2.0 kg, 95% CI: − 3.3 to − 0.7 kg, *p* = 0.004; CON: − 2.8 kg, 95% CI: − 3.8 to − 1.7 kg, *p* < 0.001). The amount of weight loss did not differ significantly between groups (Fig. 2a) but reductions in waist circumference were significantly greater in the HIIT group compared to CON (*p* = 0.001, Fig. 2b). Group-specific pre- and post-intervention values are presented in Table 2.

**Table 2:** Anthropometric and body composition data pre and post-intervention.

Variable	HIIT group (n = 32)		MIIT group (n = 22)		CON group (n = 33)	
	Pre	Post	Pre	Post	Pre	Post
Weight (kg)	116.7 ± 28.1	112.7 ± 27.6***	101.1 ± 18.1	99.1 ± 18.7**	106.4 ± 20.2	103.6 ± 20.9***
BMI (kg/m^2^)	38.5 ± 6.8	37.1 ± 6.8***	35.7 ± 5.0	34.9 ± 4.9**	37.5 ± 5.4	36.5 ± 5.8***
Fat mass (kg)	50.4 ± 15.7	46.7 ± 15.1***	44.3 ± 10.0	42.9 ± 10.1*	47.9 ± 11.7	46.1 ± 13.0**
Fat mass (%)	42.9 ± 7.5	41.1 ± 7.8***	44.0 ± 7.0	43.5 ± 6.9	45.2 ± 7.0	44.4 ± 7.7*
Fat free mass (kg)	66.3 ± 16.7	66.0 ± 17.0	56.8 ± 13.0	56.2 ± 13.0	58.3 ± 13.6	57.5 ± 13.3**
Total body water (L)	49.4 ± 12.3	49.1 ± 12.4	42.5 ± 9.3	41.9 ± 9.2*	43.8 ± 9.8	43.0 ± 9.0
Waist (cm)	117.2 ± 20.0	110.8 ± 17.9***	109.0 ± 12.4	105.3 ± 12.1**	110.3 ± 12.3	109.3 ± 13.3

Values are expressed as mean ± SD. *HIIT* high-intensity interval training group, *MIIT* moderate-intensity interval training group, *CON* control group, *BMI* body mass index. *(*p* < 0.05), **(*p* < 0.01), ***(*p* < 0.001), significantly different from pre-intervention.

### Nutritional analysis

Ten participants (HIIT: n = 4, MIIT: n = 2, CON: n = 4) missed to provide a complete follow-up dietary record and were thus not included in the nutritional evaluation. These participants did not differ significantly from those who provided complete follow-up records in respect to exercise adherence or any outcome variables examined. The assumption that missing data were completely at random was verified by a non-significant Little's MCAR test (*p* = 0.980).

Significant main effects of time were found for energy (*p* = 0.001, ή^2^ = 0.14), fat (*p* = 0.003, ή^2^ = 0.11) and carbohydrate intakes (*p* = 0.001, ή^2^ = 0.13). Subsequent post hoc tests revealed that only the CON group achieved a statistically significant reduction in total energy intake (− 463 kcal/day, 95% CI: − 854 to 73 kcal/day, *p* = 0.022). Group-specific nutritional intakes are shown in Table 3.

**Table 3:** Daily nutritional intake pre-intervention and during the last week of the intervention.

Variable	HIIT group (n = 28)		MIIT group (n = 20)		CON group (n = 29)	
	Pre	Post	Pre	Post	Pre	Post
Total energy (kcal/d)	2449 ± 1050	2035 ± 833	2036 ± 713	1793 ± 745	2259 ± 824	1795 ± 738*
Total protein (g/d)	96 ± 44	95 ± 37	80 ± 28	78 ± 27	94 ± 30	84 ± 33
Relative protein (g/kg/d)	0.9 ± 0.4	0.9 ± 0.3	0.8 ± 0.3	0.8 ± 0.3	0.9 ± 0.4	0.9 ± 0.4
Total fat (g/d)	96 ± 42	80 ± 41	84 ± 40	76 ± 36	96 ± 49	72 ± 40*
Relative fat (g/kg/d)	0.8 ± 0.4	0.7 ± 0.3	0.9 ± 0.5	0.8 ± 0.4	0.9 ± 0.5	0.7 ± 0.4
Total CHO (g/d)	224 ± 85	208 ± 97	206 ± 72	172 ± 86*	224 ± 90	180 ± 79*
Relative CHO (g/kg/d)	2.0 ± 0.8	1.9 ± 0.7	2.0 ± 0.7	1.8 ± 0.9	2.2 ± 1.0	1.8 ± 0.8

Values are expressed as mean ± SD. *HIIT* high-intensity interval training group, *MIIT* moderate-intensity interval training group, *CON* control group, *CHO* carbohydrates. *(*p* < 0.05), significantly different from pre-intervention.

### Training data

Recorded heart rate (HR) values during all exercise sessions confirmed that the prescribed level of intensity was achieved in both exercise groups. Average HR reached at the end of each interval bout was 93 ± 5% of HR_max_ in the HIIT group and 79 ± 5% of HR_max_ in the MIIT group, respectively. The adherence rates (the percentage of the scheduled training sessions that the participants completed) in both exercise groups were high (HIIT group: 94.6 ± 7.6% and MIIT group: 91.7 ± 8.6%). No adverse events occurred at any point during the training sessions.

### Cardiorespiratory fitness and physical performance

The average baseline VO_2max_ (20.9 ± 5.3 mL/kg/min) indicated that the CRF level was generally very poor in the total sample. Two participants from the CON group were not able to perform the post-intervention cycle ergometer test due to an injury unrelated to the study and thus, the final sample size for this group consisted of 31 participants. A significant group-by-time interaction and main effect of time was observed for absolute VO_2max_ (*p* < 0.001, ή^2^ = 0.38 and *p* < 0.003, ή^2^ = 0.10, respectively), relative VO_2max_ (*p* < 0.001, ή^2^ = 0.36 and *p* < 0.001, ή^2^ = 0.27, respectively), absolute W_max_ (*p* < 0.001, ή^2^ = 0.52 and *p* < 0.001, ή^2^ = 0.33, respectively), relative W_max_ (*p* < 0.001, ή^2^ = 0.44 and *p* < 0.001, ή^2^ = 0.41, respectively) and power output at VT (W_VT_, *p* < 0.001, ή^2^ = 0.40 and *p* < 0.001, ή^2^ = 0.37, respectively). A significant main effect of group was found for absolute VO_2max_ (*p* < 0.014, ή^2^ = 0.10) and W_max_ (*p* < 0.034, ή^2^ = 0.08). The HIIT group achieved significantly greater improvements in absolute VO_2max_ compared to MIIT and CON and a greater increase in relative VO_2max_ compared to CON (Fig. 2c,d). Moreover, the HIIT group achieved significantly greater increases in absolute W_max_ (vs. MIIT: 17 W, 95% CI: 9 to 26 W, *p* < 0.001; vs. CON: 30 W, 95% CI: 22 to 37 W, *p* < 0.001) and W_VT_ (vs. MIIT: 18 W, 95% CI: 8 to 27 W, *p* < 0.001; vs. CON: 28 W, 95% CI: 20 to 36 W, *p* < 0.001). In the MIIT group, improvements in VO_2max_ (Fig. 2c,d), W_max_ (12 W, 95% CI: 4 to 20 W, *p* = 0.002) and W_VT_ (11 W, 95% CI: 2 to 20 W, *p* = 0.015) were significantly greater compared to CON. Pre- and post-intervention values for each group are shown in Table 4.

**Table 4:** Cardiorespiratory fitness and exercise performance variables pre- and post-intervention.

Variable	HIIT group (n = 32)		MIIT group (n = 22)		CON group (n = 31)	
	Pre	Post	Pre	Post	Pre	Post
VO_2max_ (L)	2.51 ± 0.69	2.75 ± 0.68***^a^	2.06 ± 0.56	2.12 ± 0.55	2.24 ± 0.87	2.09 ± 0.92**
VO_2max_ (mL/kg/min)	21.9 ± 5.3	25.1 ± 5.4***^aa^	20.3 ± 3.8	21.5 ± 4.3*	21.0 ± 7.3	20.7 ± 7.8
W_max_ (W)	158 ± 46	182 ± 43***^bb^	133 ± 40	140 ± 43*	156 ± 62	150 ± 61*
W_max_ (W/kg)	1.4 ± 0.4	1.7 ± 0.4***	1.3 ± 0.3	1.4 ± 0.3**	1.5 ± 0.6	1.5 ± 0.6
W_VT_ (W)	58 ± 27	84 ± 28***^b^	53 ± 24	62 ± 29**	68 ± 32	66 ± 30
HR_max_ (b/min)	160 ± 18	161 ± 18	153 ± 22	153 ± 24	155 ± 24	150 ± 24

Values are expressed as mean ± SD. *HIIT* high-intensity interval training group, *MIIT* moderate-intensity interval training group, *CON* control group, *VO*_*2max*_ maximal oxygen uptake, *W*_*max*_ maximal power output, *W*_*VT*_ power output at the ventilatory threshold, *HR*_*max*_ maximal heart rate. *(*p* < 0.05), **(*p* < 0.01), ***(*p* < 0.001), significantly different from pre-intervention. ^a^(*p* < 0.05), ^aa^(*p* < 0.01), significant difference HIIT vs. CON; ^b^(*p* < 0.05), ^bb^(*p* < 0.01), significant difference HIIT vs. MIIT and CON.

### Cardiometabolic risk markers

A significant group-by-time interaction and main effect of time was detected for systolic blood pressure (*p* = 0.001, ή^2^ = 0.15 and *p* < 0.001, ή^2^ = 0.21, respectively), diastolic blood pressure (*p* = 0.006, ή^2^ = 0.11 and *p* < 0.001, ή^2^ = 0.20, respectively), MAB (*p* = 0.001, ή^2^ = 0.15 and *p* < 0.001, ή^2^ = 0.24, respectively) and MetS z-score (*p* < 0.027, ή^2^ = 0.08 and *p* < 0.001, ή^2^ = 0.20, respectively). Both, the HIIT and MIIT group showed greater reductions in blood pressure values from pre- to post-intervention than CON (Fig. 2e). Reductions in the MetS z-score were significantly greater in the HIIT group compared to CON (Fig. 2f). Group-specific pre- and post-intervention values of all cardiometabolic risk markers are shown in Table 5.

**Table 5:** Cardiometabolic risk variables pre- and post-intervention.

Variable	HIIT group (n = 32)		MIIT group (n = 22)		CON group (n = 33)	
	Pre	Post	Pre	Post	Pre	Post
MetS z-score	3.30 ± 3.79	1.48 ± 3.10***	2.77 ± 3.93	1.58 ± 3.85**	1.79 ± 2.92	1.25 ± 2.77
Systolic BP (mmHg)	144 ± 16	133 ± 11***	143 ± 14	131 ± 11**	136 ± 15	136 ± 12
Diastolic BP (mmHg)	94 ± 11	86 ± 7***	90 ± 7	85 ± 8*	86 ± 11	86 ± 9
MAB (mmHg)	110 ± 11	102 ± 7***	108 ± 8	100 ± 8**	103 ± 11	103 ± 9
Glucose (mg/dL)	101 ± 17	101 ± 14	106 ± 29	109 ± 32	100 ± 18	97 ± 17
HbA_1c_ (%)	5.7 ± 0.5	5.6 ± 0.3	5.8 ± 0.9	5.8 ± 0.9	5.6 ± 0.7	5.5 ± 0.6
Triglycerides (mg/dL)	137 ± 59	133 ± 45	151 ± 86	143 ± 88	151 ± 80	128 ± 61
Cholesterol (mg/dL)	219 ± 33	218 ± 37	202 ± 41	203 ± 37	227 ± 47	217 ± 37
HDL (mg/dL)	48 ± 10	49 ± 11	51 ± 14	51 ± 12	54 ± 12	54 ± 13
LDL (mg/dL)	148 ± 28	147 ± 31	129 ± 27	131 ± 27	146 ± 33	142 ± 29
LDL/HDL ratio	3.2 ± 1.0	3.0 ± 0.9	2.6 ± 0.7	2.7 ± 0.7	2.8 ± 0.8	2.8 ± 0.7

Values are expressed as mean ± SD. *HIIT* high-intensity interval training group, *MIIT* moderate-intensity interval training group, *CON* control group, *BP* blood pressure, *MAB* mean arterial blood pressure, *HbA*_*1c*_ glycosylated hemoglobin A_1c_, *HDL* high-density lipoprotein cholesterol, *LDL* low-density lipoprotein cholesterol. *(*p* < 0.05), **(*p* < 0.01), ***(*p* < 0.001), significantly different from pre-intervention.

### Self-reported outcomes

There were significant changes in self-reported QoL over time (*p* < 0.001, *W* = 0.18). Group-specific analyses showed significant improvements in the HIIT (+ 10%, 95% CI: 4 to 16%, *p* < 0.001) and MIIT group (+ 8%, 95% CI: 0 to 16%, *p* = 0.029) on the EQ VAS scale. No significant changes were observed in the CON group. The average enjoyment of the exercise protocol was significantly higher in the HIIT group compared to the MIIT group (6.1 ± 0.7 points vs. 5.3 ± 0.9 points on a 7-point rating scale, *p* = 0.001). Likewise, a higher proportion of participants from the HIIT group (97%) reported that they intended to further engage regularly in this specific exercise protocol after termination of the study compared to the MIIT group (58%). Group-specific values are presented in Table 6.

**Table 6:** Self-reported health-related quality of life pre- and post-intervention.

Variable	HIIT group (n = 32)		MIIT group (n = 22)		CON group (n = 33)	
	Pre	Post	Pre	Post	Pre	Post
EQ5DL5 Index	0.867 ± 0.14	0.883 ± 0.16	0.842 ± 0.15	0.868 ± 0.14	0.859 ± 0.15	0.790 ± 0.26
EQ VAS (%)	65 ± 16	75 ± 18***	65 ± 21	73 ± 18*	58 ± 23	62 ± 27
Exercise enjoyment (0–7)^1^	–	6.1 ± 0.7^aa^	–	5.3 ± 0.9	–	–
Intention to continue with exercise protocol^1^	–	97%	–	58%	–	–

Values are expressed as mean ± SD. *HIIT* high-intensity interval training group, *MIIT* moderate-intensity interval training group, *CON* control group, *VAS* visual analogue scale. ^1^, only assessed post-intervention. *(*p* < 0.05), ***(*p* < 0.001), significantly different from pre-intervention. ^aa^(*p* < 0.01), significant difference HIIT vs. MIIT.

## Discussion

The key findings of this study were that: (i) as little as 28 min of low-volume HIIT per week led to significant improvements in CRF and cardiometabolic risk severity in obese MetS patients, (ii) among the three study groups, improvements in VO_2max_ and MetS z-score were greatest in the HIIT group, however, low-volume MIIT was still sufficiently effective to improve CRF and cardiometabolic risk profile in obese MetS patients, (iii) caloric restriction without exercise (CON group) was helpful for the reduction of body weight, however, despite comparable amounts of weight loss in the three study groups, only participants who additionally exercised experienced beneficial changes in cardiometabolic health outcomes and perceived QoL.

It is well established that the degree of VO_2max_ is a major predictor of future CVD risk and all-cause mortality^8^ and it has been suggested that low CRF should be regarded as an independent core component of MetS^50^. More specifically, Lee et al.^51^ noted that obese individuals with a good level of CRF exhibit a lower risk of CVD and premature mortality compared to lean unfit individuals. Estimates indicate that an increase in VO_2max_ by 1 mL/kg/min is associated with a 9% reduction of cardiovascular mortality risk^52^. Thus, the average increase in VO_2max_ by 3.1 mL/kg/min (~ 15%) observed in the HIIT group can be considered highly clinically significant. In addition, we assessed W_VT_, a submaximal marker of CRF, which is more specific to determine the ability to perform physical activities of daily living^46^. The significant increase in W_VT_ by 26 W (~ 30%) following HIIT may indicate metabolic adaptations (e.g. improved mitochondrial function) that are linked with enhanced capacity to perform sustained submaximal activities^53^.

To date, there are limited studies investigating the effects of low-volume HIIT in obese individuals at increased cardiometabolic risk. Comparable to our results, Madsen et al.^30^ observed that 8 weeks of low-volume HIIT (10 × 1 min at 90% HR_max,_ 3 weekly sessions) led to significant improvements in VO_2max_ by 3.0 mL/kg/min (~ 13%) in obese type 2 diabetes patients. Ramos et al.^27^ reported that VO_2max_ increased by 1.8 mL/kg/min (~ 6%) after 16 weeks of low-volume HIIT in obese individuals diagnosed with MetS. Another recent study^31^ found only modest changes in VO_2max_ (0.8 mL/kg/min, ~ 4%) following 12 weeks of low-volume HIIT (1 × 4 min at 90% VO_2max_, 3 sessions/week) in individuals with obesity and type 2 diabetes. The differential effects on CRF that were observed in the current and previous studies could be related to differences in the specific type of the applied HIIT protocol. It has been suggested, for example, that multiple shorter intervals might provide a more effective stimulus for adaptations in the cardiovascular system than fewer intervals with longer duration in low-volume HIIT protocols^54^. This assumption is supported by the finding that increases in VO_2max_ were greater in our study (5 × 1 min HIIT) and that of Madsen et al.^30^ (10 × 1 min HIIT) compared to those observed in the studies that have applied only a single 4 min bout of HIIT^27,31^. At present, though, this remains speculative, as the influence of differing interval durations on CRF and other health outcomes is still insufficiently investigated and remains to be further explored in future studies. However, it is important to emphasize that the time effort for our HIIT protocol (28 min/week) was ~ 45–65% lower than in the previous studies with weekly exercise volumes ranging from 51 to 113 min (including warm-up and cool-down phases)^27,28,30,31^. Given that “lack of time” is one of most commonly cited barriers to maintaining a more physically active lifestyle in obese individuals^10^, it is a crucial finding of our study that further reductions in the total volume of HIIT are still effective for improvements in CRF in obese MetS patients, which may be an important factor for longer-term adherence to exercise.

Moreover, we observed a significant mean reduction in MetS z-score by − 1.8 units following HIIT, which is comparable to the findings of Ramos et al.^27^ (mean reduction: − 1.6 units). Collectively, these findings demonstrate that targeted low-volume HIIT interventions may improve cardiometabolic health effectively and at least to a similar extent as traditional high-volume MICT programs^43^, despite substantial lower time effort. In line with previous research^27^, the MetS z-score reduction was in large part due to a significant improvement in blood pressure. It is well established that elevated blood pressure increases the risk of CVD morbidity and mortality^55^ and it has been reported that that every blood pressure reduction of 10 mmHg systolic or 5 mmHg diastolic lowers risk of CVD and stroke by 22% and 41%, respectively^56^. Additionally, it has been reported that a 10% reduction in waist circumference corresponds to a ~ 1.5 times lower mortality risk^57^. Thus, the observed average reductions in systolic (~ 11 mmHg) and diastolic (~ 8 mmHg) blood pressure and waist circumference (~ 6%) in the HIIT group are therefore very likely to provide clinically relevant benefits, comparable to effects obtained in pharmacological studies^55,56^. In contrast to previous low-volume HIIT-studies in individuals at increased cardiometabolic risk, reporting beneficial effects on fasting glucose^28–30^, HbA_1c_^28,30,31^, triglycerides^28^ and HDL^27,28^, we found no significant changes in blood markers of glucose and lipid metabolism. It might be speculated, therefore, that the total energy expenditure from our extremely low-volume HIIT protocol may have been too low to induce positive alterations in participants’ glycemic and lipid profiles. However, we have previously observed significant reductions in LDL levels following 8 weeks of our low-volume HIIT protocol in normal-weight sedentary individuals^34^ and another study, applying brief “all-out” SIT (30 min time effort per week) in sedentary men, found improved glycaemic control after a 12-week intervention period^58^. Given that participants in our previous study^34^ and that of Gillen et al.^58^ were normal-weight to slightly overweight but otherwise healthy individuals, it might also be conceivable that the insufficient improvements in blood lipid and glucose profiles in the present investigation may be due to pre-existing less favorable metabolic conditions in our obese MetS patients. Previous research, which indicated altered substrate utilization during exercise in obese compared normal-weight individuals^59,60^, may hint into this direction. Thus, further very low-volume HIIT studies are needed to draw more comprehensive conclusions on this issue.

In accordance with previous research in MetS patients^27^, no adverse events occurred in the present study, which indicates that very low-volume HIIT consisting of relatively intense but submaximal interval bouts at intensities between 80 and 95% of HR_max_ can be safely applied to obese individuals at increased cardiometabolic risk, provided that medical clarification is carried out beforehand. Importantly, our data indicate that the applied HIIT protocol was also well accepted by participants, as became evident by high ratings of exercise enjoyment and the fairly low number of dropouts (20%) when compared to other obesity interventions^37^. It is widely recognized that feelings of pleasure and enjoyment are crucial factors for adherence to exercise programs^61^. Although it has been argued that untrained individuals typically tend to avoid participating in strenuous exercise and thus, one would expect that HIIT could elicit negative psychological responses^32^, there is growing evidence that HIIT seems to be more enjoyable than MICT in overweight and obese individuals^61,62^. Moreover, a recent meta-analysis from our group has demonstrated that exercise intensity (in contrast to exercise volume) was not related to dropout from HIIT interventions in previously sedentary individuals^63^.

However, although our and previous data^27^ indicate that HIIT appears to be well tolerated and accepted by high-risk populations, high-intensity exercise may not be applicable to some patients due to particular medical contraindications or lack of motivation, requiring the development and evaluation of alternative, less intense interval training regimes. To date, research elucidating the impact of MIIT on cardiometabolic health, particularly in obese individuals, is still very scarce^64^. Racil et al.^65^ observed a mean increase in VO_2max_ by ~ 3.6% and significant reductions in systolic and diastolic blood pressure by 4 mmHg respectively, in obese adolescent females following 12 weeks of MIIT (3 weekly sessions consisting of short running bouts at 80% of maximal aerobic speed). More recently, Nazari et al.^66^ reported that 8 weeks of MIIT (30 s cycle ergometer bouts at 75–80% HR_max_, 3 sessions/week) improved blood lipid profile in overweight/obese women. These initial studies have provided first evidence that MIIT may have beneficial impact on health outcomes in individuals with obesity, however, given that the total time effort ranged from ~ 120 to ~ 180 min per week in these trials, the timesaving is quite small compared to the traditional exercise programs.

To the best of our knowledge, the present study was the first to investigate whether a simultaneous reduction in interval training volume and intensity (i.e. low-volume MIIT) would still be effective at improving CRF and related health-outcomes in obese individuals. As hypothesized, the beneficial effects on VO_2max_ and MetS z-score were less pronounced than those achieved with HIIT. This finding is in accordance with previous research, suggesting that the improvement in VO_2max_ is intensity-dependent^58,67^ and over the long term, it has been shown that participation in regular vigorous exercise is associated with a greater long-term risk reduction in morbidity and all-cause mortality than moderate-intensity exercise^68^. The greater reductions in waist circumference and fat mass in the HIIT group compared to the MIIT group are also consistent with recent meta-analyses, indicating that higher exercise intensity may be more effective in improving body composition^15,18^. The potential underlying mechanisms for the superior effects of higher exercise intensity on body fat mass include higher energy expenditure during exercise^69^, increased catecholamine and growth hormone release^70,71^ and greater post-exercise oxygen uptake^72^ compared to lower exercise intensity. Moreover, it has been reported that exercise intensity may differentially affect physiological mechanisms involved in appetite regulation. While low- and moderate-intensity exercise appear to have little impact on appetite perception^73^, it has been demonstrated that high-intensity exercise may have appetite-suppressing effects, which were associated with exercise-induced changes in peripheral gut hormones like ghrelin^74^ and modifications in brain receptors controlling central appetite regulation^75^. The observed greater (although not statistically significant) reduction of energy intake in the HIIT group compared to the MIIT group supports these previous findings. However, there is still lack of evidence whether HIIT actually leads to lower energy intake over the longer term when compared to moderate-intensity exercise^76^.

Interestingly, the HIIT group also reported greater exercise enjoyment than the MIIT group, which could be attributed to differential perceptions of improvements in fitness, health status or body image. It has been reported, for example, that perceived health improvement (including weight loss rates) is significantly associated with participants’ satisfaction in weight loss programs^77^. Thus, it is conceivable that greater/faster training progress contributed to higher enjoyment and finally also led to the considerably lower dropout rate in the HIIT group (20%) compared to the MIIT group (40%). However, it should be highlighted that a 1.2 mL/kg/min increase in VO_2max_ and a reduction in systolic (~ 12 mmHg) and diastolic (~ 5 mmHg) blood pressure in the MIIT group can be considered clinically significant^52,55,56^. Thus, low-volume MIIT might be a viable exercise option for individuals who are not able or willing to engage in more strenuous exercise or used as an initial preparatory training step prior to HIIT. From a practical point of view, these results provide evidence that even very small amounts of exercise performed at moderate intensity can have beneficial impact on health outcomes in populations at increased cardiometabolic risk.

Apart from physiological benefits, it is well established that regular exercise can also improve parameters of mental health and well-being^78^. However, the effects of low-volume HIIT on psychological outcomes have only scarcely been investigated. In accordance with the literature linking obesity with diminished QoL^79^, we found that our participants exhibited average baseline EQ-VAS scores (58–65%) that were substantially lower compared to those reported for the general population (~ 72%)^47^. Post-intervention results revealed that both HIIT and MIIT produced improvements in EQ-VAS by 10% and 8%, respectively, that have been considered clinically meaningful^80^. In contrast to EQ-VAS, EQ-5Q-L5 index only tended to increase in both exercise groups but did not reach significance, although both measures were administered within the same questionnaire and at the same time. At first glance, this seems surprising, however, according to previous research, differences between these two measures are not uncommon and may be attributed to their specific scoring mechanisms^81^. More specifically, EQ-5Q-L5 index measures five common dimensions of health-related QoL, which are converted into a single score, while EQ-VAS asks respondents to rate their overall health (potentially including aspects not covered by the five EQ-5Q-L5 dimensions). Consequently, respondents with a maximum possible EQ-5Q-L5 score may provide an EQ-VAS rating that is less than the full score of 100, and vice versa.

Within the 12-week intervention period, all three groups achieved a significant reduction of body weight ranging from 2.0% (MIIT group) to 3.4% (HIIT group), which is in accordance with average weight loss amounts observed in most obesity programs^82^. It is commonly suggested, however, that a weight loss of at least 5% should be achieved to produce clinically meaningful improvements in cardiometabolic risk factors, like blood pressure, blood lipids or glucose, and this threshold is also often used to define a “successful” obesity treatment^83^. In line with this, the lack of significant changes in any cardiometabolic risk outcome in the CON group may indicate that the amount of weight loss was too small to promote improvements in cardiometabolic health. In this context, however, it is important to point out that although there were no significant differences in the magnitude of total body weight reduction between groups, it was notable that waist circumference (as surrogate measure of abdominal visceral adipose tissue) significantly decreased following HIIT and MIIT, contrary to the control group. This finding is consistent with previous research, suggesting that parallel but opposing changes in body fat mass and lean mass may occur in response to increased PA that cannot be detected by body weight changes but by waist circumference^84^. In line with this, fat free mass was maintained in both exercise groups, whereas in the CON group, fat free mass decreased significantly following weight loss. Furthermore, and even more importantly, weight loss without exercise failed to produce a meaningful reduction in blood pressure and resulted in a significant decrease in VO_2max_. The observed reduction of VO_2max_ in the CON group is in line with a previous study, reporting a 6% decrease in aerobic capacity in overweight women, who lost 7% of body weight during 16 weeks of caloric restriction without additional exercise^85^. The underlying physiological mechanisms responsible for the deterioration of CRF after weight loss from caloric restriction are not yet fully understood but may be related to skeletal muscle atrophy, catabolic processes in the cardiovascular system^85^ and unfavorable changes in endocrine and haemotopoietic systems^86^. Given the significant association between CRF and health-related QoL^87^, it is not surprising that only the two exercise groups perceived significant improvements in subjective health status. These findings support previous research^84^, suggesting that body weight reduction should not be regarded as the only and major indicator of a “successful” obesity treatment, and that improving CRF should be a key therapeutic target for cardiometabolic risk reduction in obese patients.

There are some limitations of this study that should be considered. First, given that the intervention period was completed after 12 weeks, the long-term efficacy of very low-volume HIIT/MIIT (including long-term adherence) remain to be investigated in obese populations and individuals at increased cardiometabolic risk. Larger-scale, preferably multicenter studies, involving long-term intervention periods will be needed to answer such questions. Second, it must be considered that the present study was conducted in a well-controlled setting with careful supervision of all exercise sessions. Thus, further research will be needed to clarify whether obese individuals at increased cardiometabolic risk would be able and/or willing to conform with the present exercise protocols without a close supervision. Future studies may also wish to concomitantly compare low-volume HIIT, MIIT and MICT (which is still one the most commonly prescribed exercise modalities in obesity treatment) in obese populations within one trial in order to investigate more specifically the effect of training intensity (high vs. moderate) and stimulus type (intermittent vs. continuous) on cardiometabolic health. Such efforts would be helpful to further elucidate the value of low-volume MIIT as an alternative exercise option in clinical settings.

In conclusion, this study demonstrates that less than 30 min of interval training per week, corresponding to only one fifth of the generally recommended minimum amount of PA, may induce clinically relevant positive changes in CRF and cardiometabolic health as well as significant improvements in QoL in obese MetS patients. Moreover, our findings suggest that very low-volume interval training, even when done at moderate intensity (i.e. < 80% HR_max_), is still sufficiently effective to induce beneficial health effects that go far beyond simple weight loss through caloric restriction alone.

## Data Availability

The datasets generated and analyzed during the current study are not publicly available but are available from the corresponding author on reasonable request.
